# To Improve Higher Trainees' Experience With Out of Hour (OOH) Working Through Local Induction Programme

**DOI:** 10.1192/bjo.2024.312

**Published:** 2024-08-01

**Authors:** Ting Miller, Chandrashekar Natarajan, Ismail Laher

**Affiliations:** 1 Leeds and York Partnerships NHS Foundation Trust (LYPFT), Leeds, United Kingdom; 2 Leeds and York Partnerships NHS Foundation Trust, Leeds, United Kingdom

## Abstract

**Aims:**

Out-of-hours (‘on-call') work can be perceived as daunting by junior doctors. When psychiatry trainees progress from core trainee to higher trainee, what entails ‘on-call' work often shifts dramatically. Current allocation policy in Yorkshire and Humber Deanery means most of the higher trainees (HTs) begin their first on-call as a HT in a trust where they have never worked before. This frequently entails navigating an unfamiliar patient record system and various OOH care pathways in a new work environment, which can make the first few on-call shifts extremely stressful and potentially increase the risk of clinical errors.

We aim to evaluate the on-call experiences among higher trainees, collect feedback on ways of improving induction programme relating to OOH work and re-evaluation after the interventions implemented in the latest induction.

**Methods:**

- A short survey using Likert scale was designed to capture HTs' experience and knowledge in relation to OOH work plus free text feedback at the end of each question.
- An online survey link was disseminated by email in May 2023 among HTs who joined LYPFT between August 2022 to Feb 2023.
- Interventions: a) A face to face induction in August 2023 to replace the online induction; b) ‘A walkabout tour at Crisis office’ led by Crisis consultant as part of the induction programme.
- Re-survey link was sent out in October 2023 to HTs who joined in August 2023.

**Results:**

1st Survey: 11 out 16 new HTs completed the survey. 5 out of 11 had never worked in LYPFT.

2nd Survey: 11 out of 19 new HTs completed the survey. 8 out of 11 had never worked in LYPFT.

2nd Survey showed significant improvement in HTs' level of familiarity to on-call office environment, awareness of the multi-agency S136 pathway and local policy as well as alternative local crisis provisions other than hospital admission, and the relevant referral procedures. HTs' confidence of navigating OOH local care pathway was markedly enhanced.

Overwhelming positive feedback were received regarding the ‘Walkabout tour' as part of the Induction programme.

**Conclusion:**

- Simple interventions at Induction programme can significantly improve HTs' confidence for OOH work.
- HTs valued high on practical support such as the ‘Walkabout tour at Crisis office' and would like it to be expanded to other OOH services such as Seclusion unit and Acute Liaise Psychiatry Service.

